# Using wearable sensors to classify subject-specific running biomechanical gait patterns based on changes in environmental weather conditions

**DOI:** 10.1371/journal.pone.0203839

**Published:** 2018-09-18

**Authors:** Nizam Uddin Ahamed, Dylan Kobsar, Lauren Benson, Christian Clermont, Russell Kohrs, Sean T. Osis, Reed Ferber

**Affiliations:** 1 Faculty of Kinesiology, University of Calgary, Calgary, Alberta, Canada; 2 Running Injury Clinic, University of Calgary, Calgary, Alberta, Canada; 3 Faculty of Nursing, University of Calgary, Calgary, Alberta, Canada; University of Illinois at Urbana-Champaign, UNITED STATES

## Abstract

Running-related overuse injuries can result from a combination of various intrinsic (*e*.*g*., gait biomechanics) and extrinsic (*e*.*g*., running surface) risk factors. However, it is unknown how changes in environmental weather conditions affect running gait biomechanical patterns since these data cannot be collected in a laboratory setting. Therefore, the purpose of this study was to develop a classification model based on subject-specific changes in biomechanical running patterns across two different environmental weather conditions using data obtained from wearable sensors in real-world environments. Running gait data were recorded during winter and spring sessions, with recorded average air temperatures of -10° C and +6° C, respectively. Classification was performed based on measurements of pelvic drop, ground contact time, braking, vertical oscillation of pelvis, pelvic rotation, and cadence obtained from 66,370 strides (~11,000/runner) from a group of recreational runners. A non-linear and ensemble machine learning algorithm, random forest (RF), was used to classify and compute a heuristic for determining the importance of each variable in the prediction model. To validate the developed subject-specific model, two cross-validation methods (one-against-another and partitioning datasets) were used to obtain experimental mean classification accuracies of 87.18% and 95.42%, respectively, indicating an excellent discriminatory ability of the RF-based model. Additionally, the ranked order of variable importance differed across the individual runners. The results from the RF-based machine-learning algorithm demonstrates that processing gait biomechanical signals from a single wearable sensor can successfully detect changes to an individual’s running patterns based on data obtained in real-world environments.

## Introduction

Running is one of the most common recreational activities around the world but despite its popularity, each year approximately 50% of runners experience a running-related musculoskeletal injury [1–3]. The etiology of overuse running injuries is multifactorial, and can result from the interaction of many extrinsic factors, such as environmental conditions, running surface, footwear, and weekly training mileage, as well as intrinsic risk factors such as age, foot strike pattern, and gait biomechanics [1–4]. Prolonged exposure to these intrinsic and extrinsic risk factors may lead to overuse running injury [5]. One risk factor that has received very little attention in the literature is whether gait biomechanical patterns change as a result of environmental weather conditions.

Previous investigations of injury risk, based on ambient temperature, have suggested that tissue damage may occur due to a lack of proper warm up. For example, Milgrom et al. reported an increased risk of Achilles paratendinitis among infantry recruits in winter conditions, as compared to summer [6]. On the other hand, cold weather has been shown to reduce shoe-surface traction, resulting in a reduced risk of acute knee and ankle injuries among football players [7, 8]. Only a handful of studies have investigated the effect of environmental weather conditions on running performance, but none have investigated whether gait biomechanics change as a result of **environmental** weather. For example, Ely et al., [9] reported a progressive reduction in marathon performance as temperatures increased from 5 to 25 degrees C, for both males and females and across competitive and recreational runners, but performance was more negatively affected for slower runners. These studies suggest that weather can affect both physiological and mechanical aspects of running gait. Thus, it is possible that different weather conditions may be associated with concomitant changes in gait biomechanical running patterns, however, to our knowledge no study has directly investigated this hypothesis.

The main reason the inter-relationship between environmental weather conditions and gait biomechanics has not been investigated is most likely due to the inability to collect such data in a laboratory setting. However, due to the availability and utility of modern portable inertial measurement units (IMUs) and global positioning **system** (GPS), it is now possible to collect data outside of the laboratory setting [10–12]. Since large quantities of data can be collected using wearable devices, machine learning (ML) techniques are also needed to better understand the complexities of gait biomechanics and how concomitant changes in biomechanical patterns may be related to injury or performance [13, 14]. Furthermore, traditional biomechanics research generally investigates potential differences between two groups using group-based analyses. For example, several researchers have identified differences in running patterns based on different age groups, gender and/or injury status [15–17]. In contrast, more recent research has shown that group-based comparisons are not efficacious due to the existence of sub-groups [18, 19], and other studies have shown that subject-specific models are necessary to understand individual differences and risk factors [20–23]. Several authors have also used different ML algorithms to develop these sub-group-based models, including principal component analysis, support vector machine and hierarchical cluster analysis [17–19]. However, to our knowledge no study has directly investigated whether a subject-specific **model** provide deeper insight into emerging IMU-based biomechanical investigations based on changes in environmental weather conditions.

Therefore, the purpose of this study was to develop a classification model based on subject-specific changes in biomechanical running patterns across two different environmental weather conditions using data obtained from wearable sensors in out-of-laboratory environments. We hypothesized that we could classify changes in subject-specific running patterns based on weather conditions with a classification accuracy greater than 80% and that the ranked order of variable importance would be based on subject-specific ML models. A secondary objective was to determine the ranking of the biomechanical variables, based on their importance in the classification margin, in order to better understand changes in subject-specific running patterns.

## Methods

### Participants

Six recreational runners (Five females: age = 47.5±9.69 years, height = 169.17±6.56 cm, weight = 67.42±11.5 kg; and one male: age = 29 years, height = 170 cm, weight = 75 kg) volunteered to participate in the study. The runners were free of any neuromuscular diseases or musculoskeletal injuries and they were registered for a half-marathon training program managed by a local running group. This protocol was approved by the University of Calgary Conjoint Health Research Ethics Board (REB 16–2035) and all runners provided their written informed consent.

### Instrumentation

**Fig 1:**
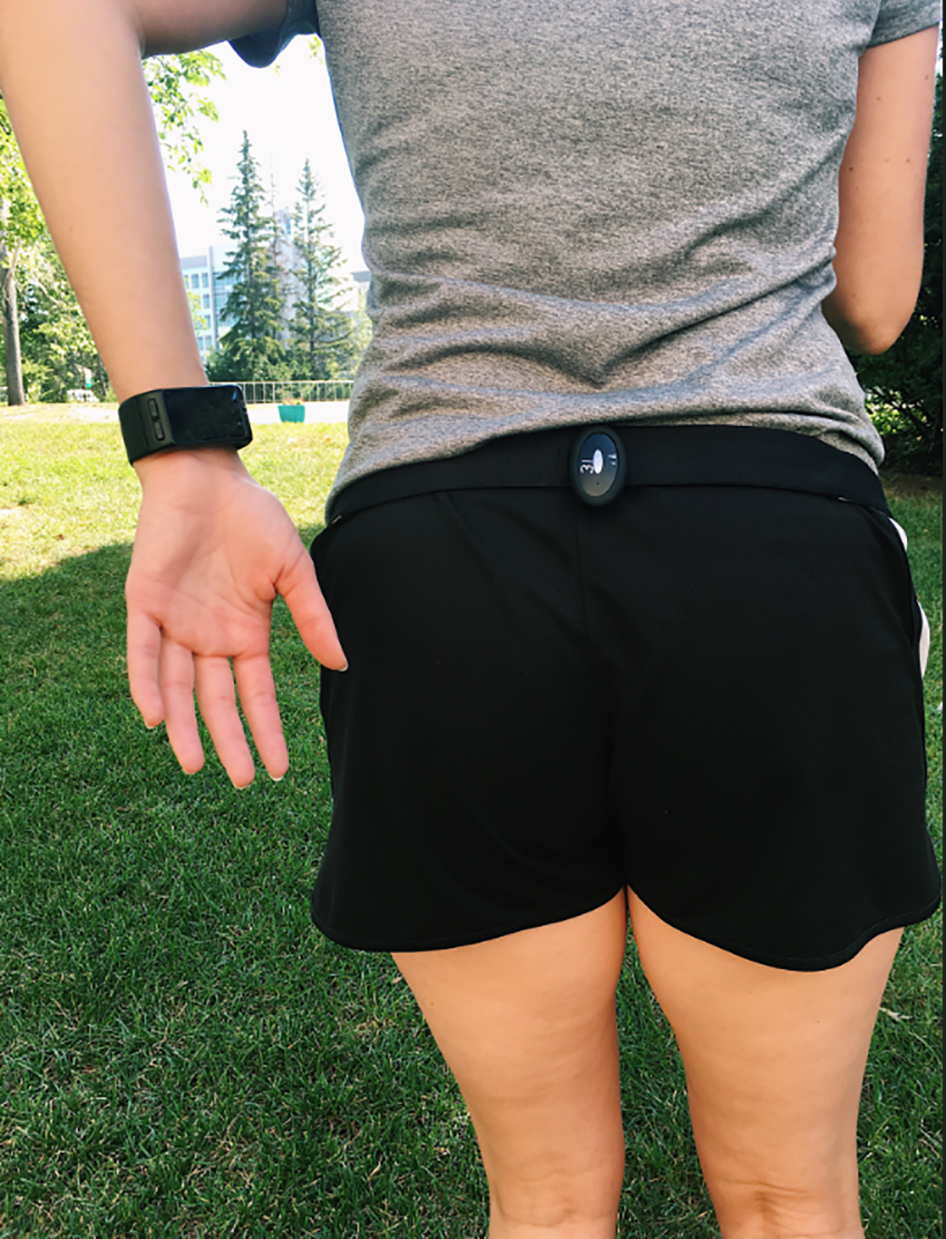
The two wearable sensors devices (Lumo Run and Garmin) used to record the data during running.

Biomechanical gait variables from each runner were recorded using the Lumo Run® (Lumo Bodytech Inc., Mountain View, CA) wearable inertial measurement unit (IMU), consisting of a 3-dimensional (3D) accelerometer, magnetometer, and gyroscope. (dimension: 4.98cm x 2.84cm x 0.99cm). The Lumo Run IMU was attached to the posterior aspect of either the runner’s waistband or running belt as per the manufacturer’s instructions [24] (Fig 1). This wearable sensor device measured and recorded data for six different biomechanical variables [24] and averaged these data for each ten-strides (Table 1) and a complete description of these variables can be found on the manufacturer’s website [24]. A GPS watch (Garmin vívoactive® HR; Garmin International Inc., KS, USA) was attached to each runner’s preferred wrist (Fig 1) and recorded running speed (m/s), distance (kilometers (km)), and global positioning data, including latitude, longitude and altitude, every second.

**Table 1:** Features recorded from the wearable devices.

Device	Features	Unit	Frequency
Lumo Run	Pelvic drop (PD) (frontal plane motion of the runner’s pelvis)	Degree (deg)	100- Hz
	Vertical oscillation of pelvis (VOP) (measurement of vertical displacement)	Centimeter (cm)	
	Ground contact time (GCT) (time of total foot contact with the ground)	Millisecond (ms)	
	Braking (reduction in forward velocity following foot strike)	Meter/sec (m/s)	
	Pelvic rotation (PR) (transverse plane motion of the runner’s pelvis)	Degree (deg)	
	Cadence (number of bilateral steps per minute)	Steps per minute (SPM)	
Garmin Vívoactive HR	Heart rate (HR)	Beats per minute (BPM)	1- Hz
	Altitude	Meter (m)	
	Distance	Kilometer (km)	
	Global position-latitude	Degree (deg)	
	Global position- longitude	Degree (deg)	
	Running speed	Meter/sec (m/s)	

### Data collection

Gait variables from winter runs were recorded from mid-February to mid-March, while spring runs were recorded from late April to mid-May. Each runner performed two training runs during each weather condition for a total of four runs used in this analysis. Each run began at 8:30 AM on a Sunday, and was completed outdoors on pavement, and along a similar route. Data corresponding to the temperature (degrees Celsius), snow depth (cm), precipitation (mm), and humidity (%) for each run were derived from three different International Air Transport Association-affiliated weather stations in Calgary, AB: Canada Olympic Park (WDU), Calgary International Airport (YYC), and Calgary INT'L CS Alberta (PCI).

For each run, data from km 0 to 1 were discarded, as this was considered a warmup period, and any data following 6-km was also not used in the analysis in order to minimize the effects of fatigue, if any. Therefore, only 5-km of data (*i*.*e*., from km 1 to 6) were analyzed from each run and in total, the input data consisted of 66,370 strides (~11,000/runner) across the four runs. Altitude, latitude and longitude data from the Garmin watch were used to ensure the elevation profile for each of the four runs were similar, and that the data from each run were collected from a route with minimal changes in elevation, in order to minimize the effect of running on uphill and/or downhill.

### Data analysis

A robust, and non-linear machine learning classifier, called Random Forest (RF), was used to develop the classification model which measured the accuracy and importance of gait biomechanical variables in classifying runs of differing environmental weather conditions. The RF classifier has been shown to provide a higher classification accuracy than other existing ML classifiers with a faster computation speed, while facilitating complex interactions among predictor variables and providing information about the importance of each predictor variable [25–27]. In other word, RF provides variable importance measures to rank predictors according to their predictive power [28]. Two validation methods (Method 1: one-against-another and Method 2: partitioning datasets) were used to ensure that the proposed RF-based subject-specific classification approach was robust and that the data were not overfit [29]. With Method 1 (one-against-another) data combining one winter run and one spring run were considered the training dataset, and the testing dataset consisted of the remaining winter and spring runs. With Method 2 (partitioning datasets), 70% of each runner’s total strides performed in both weather condition were randomly selected for training, and the remaining 30% were used for testing purposes. Individual training and test sets were generated for each subject. Each classification method was applied using the standalone Python programming language (version 3.6, www.python.org) [30]. The developed RF model was trained and cross-validated using the built-in Anaconda distribution of Python with notable packages including matplotlib, numpy, scipy, and scikit-learn (“sklearn.ensemble.RandomForestClassifier”) [31, 32]. The number of trees in the RF was set to 100, as previous research has shown this is a sufficient number for obtaining high accuracy solutions to similar classification problems [33, 34]. Additionally, the RF used a Gini index to calculate the impurity of a node from the CART (classification and regression tree) learning system in order to construct the decision trees [26]. The RF trees compute a heuristic for determining how significant a variable (6 Lumo Run gait variables) is in predicting a target (weather). Statistical analyses were performed using repeated measures ANOVA (P<0.05) and Cohen's *d* effects size estimates were calculated for each difference on the outcome measures between each weather condition.

## Results

**Fig 2:**
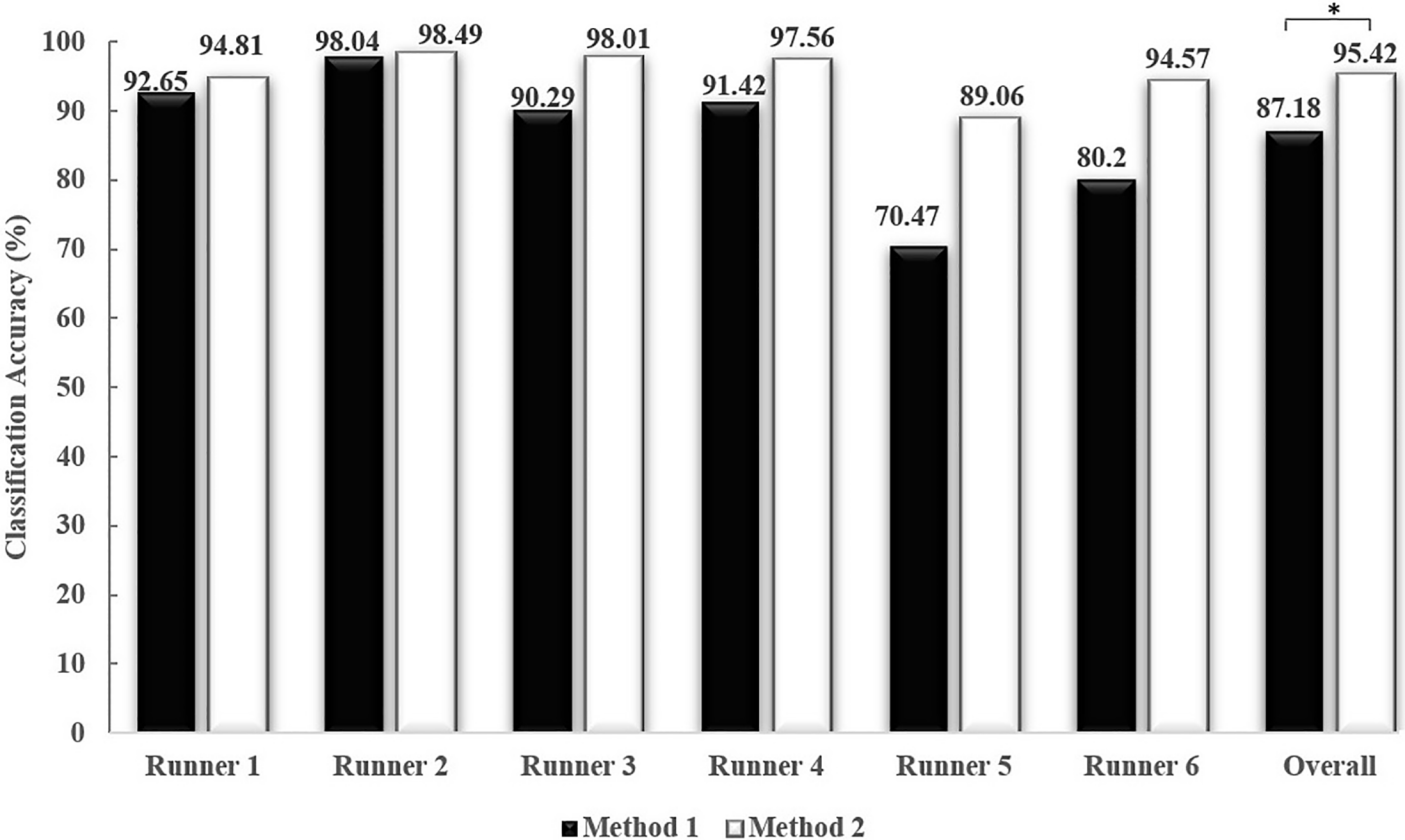
Classification accuracies obtained with Method 1 (black) and Method 2 (white); *: P<0.05.

Fig 2 presents an overview of the RF-based classification accuracy obtained with test data generated using the two validation methods. Using Method 2 (partitioning datasets), the RF-based model demonstrated an excellent overall mean classification accuracy of 95.42%. In fact, all runners yielded a classification accuracy higher than 90% with the exception of Runner 5, who exhibited a classification accuracy of 89.06%. In contrast, the overall mean classification accuracy obtained with Method 1 (one-against-another) was 87.18%, and all the runners yielded a classification accuracy higher than 85% except for Runner 5, who exhibited an accuracy of 70.47%. Significant differences (P<0.05) in the overall classification accuracies were also found between the methods. Overall, for all runners, Method 2 yielded a higher classification accuracy than Method 1. Moderate differences in classification accuracy were also observed between Methods 1 and 2 for Runner 5 (18.59%) and Runner 6 (14.37%), but the differences in classification accuracy between the methods were slight for Runner 3 (8.0%) and Runner 4 (6.14%), and non-existent for Runner 1 (2.16%), and Runner 2 (0.45%).

**Fig 3:**
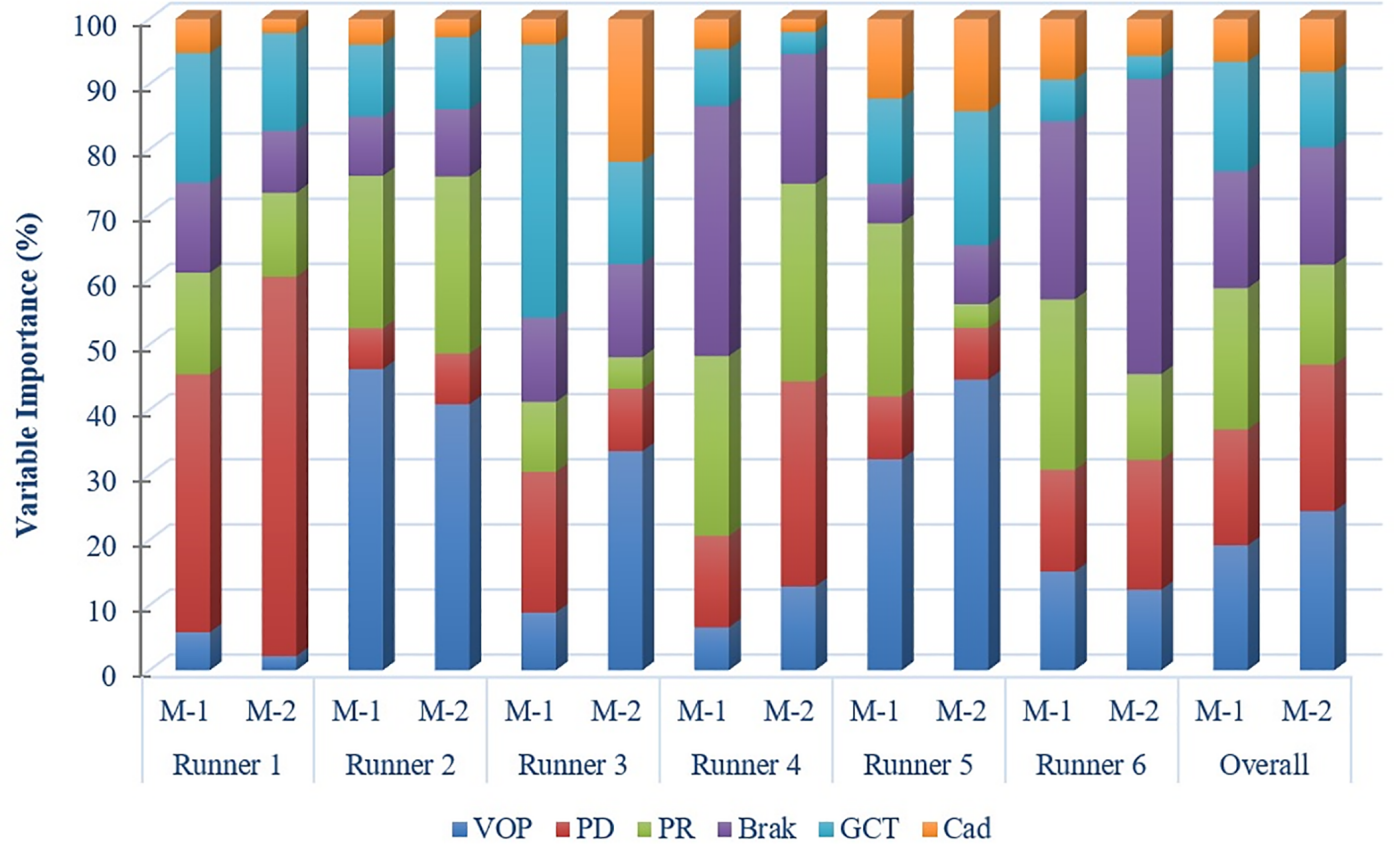
Importance of the different variables for each runner identified using two validation methods. All the variables in this stacked bar graph are shown in the same vertical order for both methods (VOP, PD, PR, braking, GCT and cadence).

**Fig 4:**
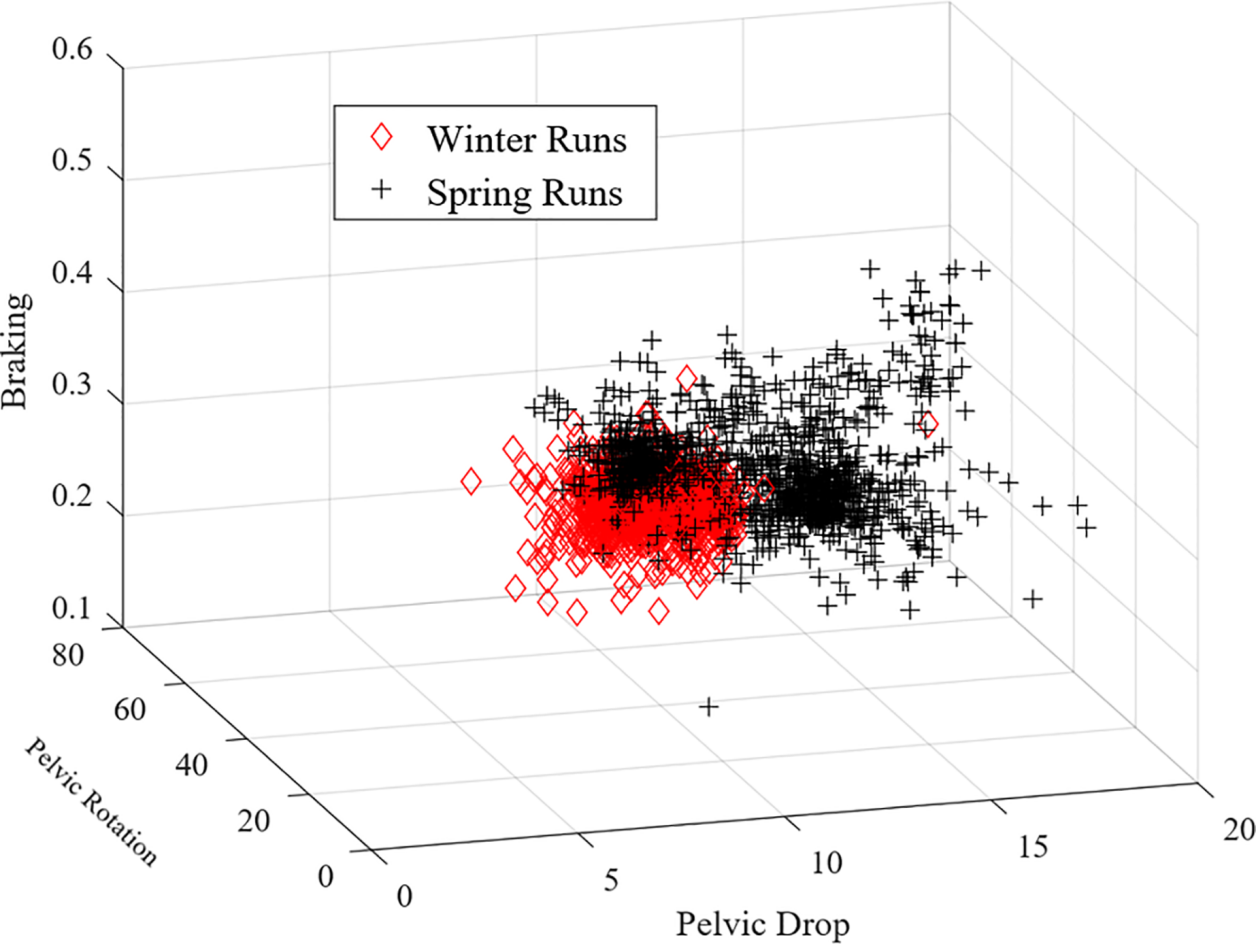
Graphical representation of the three most important variables (braking, PD and PR) for Runner 4 with Method 2. Each point is equivalent to five strides. Data from both the training and testing sets are shown.

Overall, the ranking of the variables, based on their importance in the classification margin, differed across all runners and classification methods (Table 2 and Fig 3). For example, although vertical oscillation of pelvis was the most important variable, using both methods, for Runners 2 and 5, it ranked lower for Runner 1, wherein pelvic drop was the most important variable across both methods. Similarly, pelvic rotation was the second-ranked variable for both methods for Runners 2 and 4 but was less significant for the other runners. Overall, cadence was less important for all runners, with the exception of for Runner 3, wherein it was the second most important variable using Method 2. Another notable difference was found for braking where for Runner 4 it was the most important variable using Method 1 but only the third most important variable with Method 2. A similar inconsistency was found for pelvic rotation, which was identified as the most important variable with Method 1 but was ranked fourth with Method 2. The remaining three variables, braking, ground contact time, and cadence, were not found to be important for the classification task and were consistently ranked third, fifth and sixth across both methods, respectively (Fig 4).

**Table 2:** RF-based variable importance and descriptive statistics obtained with both methods and for all individual runners.

Gait Variable	Analyzed parameters	Subject-specific results						Overall results			
		R-1	R-2	R-3	R-4	R-5	R-6	Mean±SD	95%CI	P	ES
Vertical oscillation of pelvis (cm)	M1-VI (%)	5.84	46.24	8.87	6.59	32.39	15.15	**19.18±16.53**	(-17.55, 7.07)	0.32	-0.45
	M2-VI (%)	2.18	40.84	33.64	12.87	44.63	12.37	**24.42±17.53**			
	Win (mean)	6.08	4.53	6.34	7.03	11.38	7.90	**7.21±2.33**	(-1.38, 0.21)	0.12	-0.78
	Spr (mean)	6.22	5.03	7.97	6.68	12.72	8.17	**7.81±2.68**			
Pelvic drop (deg)	M1-VI (%)	39.62	6.25	21.59	14.03	9.67	15.63	**17.80±11.91**	(-16.98, 7.62)	0.37	-0.41
	M2-VI (%)	58.23	7.78	9.57	31.46	7.91	19.93	**22.48±19.8**			
	Win (mean)	8.8	9.16	11.2	8.26	10.24	7.59	**9.21±1.32**	(-0.57, 2.89)	0.14	-0.71
	Spr (mean)	11.61	7.81	10.92	10.5	11.53	9.88	**10.37±1.41**			
Pelvic rotation (deg)	M1-VI (%)	15.57	23.41	10.74	27.62	26.55	26.15	**21.67±6.91**	(-4.35, 16.9)	0.19	0.62
	M2-VI (%)	12.9	27.17	4.85	30.38	3.66	13.17	**15.36±11.16**			
	Win (mean)	14.27	11.52	11.31	19.39	15.51	11.74	**13.96±3.16**	(-3.70, 3.89)	0.95	-0.03
	Spr (mean)	15.98	16.36	10.49	13.69	17.48	10.29	**14.05±3.09**			
Braking (m/s)	M1-VI (%)	13.85	9.1	12.91	38.42	6.1	27.33	**17.95±12.39**	(-12.50, 12.29)	0.98	-0.01
	M2-VI (%)	9.5	10.35	14.3	19.88	9.02	45.29	**18.06±13.95**			
	Win (mean)	0.27	0.25	0.36	0.34	0.3	0.31	**0.31±0.04**	(-0.03, 0.06)	0.34	-0.43
	Spr (mean)	0.27	0.22	0.36	0.37	0.31	0.4	**0.32±0.07**			
Ground contact time (ms)	M1-VI (%)	19.87	11.05	41.98	8.68	13.03	6.42	**16.84±13.15**	(-6.59, 17.15)	0.31	0.47
	M2-VI (%)	15.01	11.06	15.73	3.36	20.63	3.56	**11.56±6.97**			
	Win (mean)	258.33	254.47	298.37	247.04	290.8	272.88	**270.32±20.7**	(-7.8, 3.93)	0.43	-0.35
	Spr (mean)	267.47	263.26	297.04	243.13	290.03	272.59	**272.25±19.4**			
Cadence (steps/min)	M1-VI (%)	5.25	3.95	3.91	4.66	12.26	9.32	**6.56±3.44**	(-10.27, 7.13)	0.66	-0.19
	M2-VI (%)	2.18	2.8	21.91	2.05	14.17	5.66	**8.13±8.16**			
	Win (mean)	173.81	183.63	161.67	173.73	151.64	166.21	**168.45±11.1**	(-1.62, 7.21)	0.16	0.66
	Spr (mean)	172.53	181.29	150.68	174.48	149.05	165.89	**165.65±13.2**			

VI: variable importance; M1: Method 1; M2: Method 2; R: Runner. Win: Winter; Spr: Spring.

* P: significantly different (P<0.05)

ES: effect size (Cohen’s d). 95%CI: 95% confidence intervals

Table 2 also presents the results of the statistical analyses of the individual and overall results from both weather conditions. All runners, except Runner 4, demonstrated lower vertical oscillation of the pelvis in winter than in spring. The pelvic drop of two runners (Runner 2 and Runner 3) and the pelvic rotation of three runners (Runner 3, 4 and 6) were higher in winter than in spring. There was no clear difference in braking between winter and spring because three runners (Runners 1, 3 and 4) exhibited the same values during both conditions, two runners (Runners 4 and 6) had lower braking values in winter, and one runner (Runner 2) presented a higher braking value in winter. Two runners (Runners 1 and 2) had lower ground contact time values in winter, whereas two runners (Runners 3 and 4) had a higher ground contact time in winter, and the remaining two runners (Runners 5 and 6) had a similar value during both weather conditions. Finally, with the exception of Runner 4, all runners demonstrated a higher cadence during winter. Overall, five biomechanical variables (excluding cadence) demonstrated lower values during winter runs as compared to spring runs. However, no significant differences were found between the two weather conditions for any of the six variables (P>0.05). Cohen’s d effect size and 95% confidence intervals [95%CI] are presented in Table 2 and reveal the effect sizes between winter and spring runs were small (*i*.*e*., d<0.5), except for vertical oscillation of the pelvis, pelvic drop, and cadence, which were moderate (*i*.*e*., 0.5<d<0.8).

The results of the environmental weather conditions are presented in Table 3 and show the average temperature, humidity and snow depth were significantly different between winter and spring runs, along with no differences in precipitation.

**Table 3:** Environmental weather conditions experienced during running.

Weather	Temperature (°C)		Snow depth (cm)		Humidity (%)		Precipitation (mm)	
**Winter**	-9.74±4.85	P = 0.001 *	2.97±2.83	P = 0.001 *	75.41%	P = 0.000 *	1.35±0.89	P = 0.46
**Spring**	+5.33±2.65		0.31±0.21		63.32%		1.73±0.62	

*: Significantly different (P <0.05)

The speed and overall route were similar between sessions, as presented in Table 4. In addition, the speed, heart rate, altitude, latitude and longitude showed no significant differences between the two weather conditions (Table 4).

**Table 4:** Specific running measurements of the different runners recorded using a wearable GPS (Garmin Vívoactive HR).

Runners	Speed (m/s)		Heart rate (BPM)		Altitude (m)		Latitude (deg.)		Longitude (deg.)	
	Winter	Spring	Winter	Spring	Winter	Spring	Winter	Spring	Winter	Spring
**R-1**	2.40	2.39	161.13	154.01	1050.32	1067.18	51.05	51.05	-114.07	-114.05
**R-2**	2.36	2.36	112.45	117.97	1049.44	1071.58	50.84	51.06	-113.61	-114.06
**R-3**	2.18	2.27	146.65	143.02	1045.34	1066.30	51.05	51.06	-114.08	-114.07
**R-4**	2.39	2.36	140.68	126.82	1036.76	1061.51	51.05	51.05	-114.05	-114.05
**R-5**	2.54	2.32	154.94	155.96	1046.59	1064.64	51.05	51.06	-114.08	-114.06
**R-6**	2.36	2.40	141.99	151.14	1072.51	1051.28	51.05	51.05	-114.07	-114.05
**Overall**	**2.37**	**2.35**	**142.97**	**141.49**	**1050.16**	**1063.75**	**51.02**	**51.06**	**-113.99**	**-114.06**
	**P = 0.54**		**P = 0.57**		**P = 0.19**		**P = 0.27**		**P = 0.42**	

R: Runner.

## Discussion

The objective of this study was to classify changes in subject-specific running gait patterns based on the environmental weather (winter vs. spring) conditions using an RF classifier. The findings of the current study support our hypotheses and demonstrate that an RF-approach was a robust method for accurately classifying large datasets collected using wearable sensors in real-world settings. Interestingly, each subject’s classification method had different important predictor variables based on the RF evaluation. Therefore, each individual runner exhibited different changes in overall gait biomechanics, and changes in the weather conditions affected the mechanics of individual runners differently. To our knowledge, this study constitutes the first examination of changes in subject-specific gait biomechanics based on environmental weather conditions. These findings also support the efficacy of wearable technology, and subsequent data science approaches for understanding the complexities of running gait patterns based on collecting data in out-of-laboratory environments [29, 35].

Overall, the results of this investigation demonstrate that the presence of snow and colder temperatures results in runner-specific changes in biomechanical gait patterns, possibly in an effort to reduce the risk of falling due to the slippery surface [36]. These assumptions are supported by previous studies that also indicated injury rates were higher in colder weather conditions compared to warmer weather due to running on icy and slippery running paths [37–39]. Moreover, the results of the current study also indicate that the changes in running biomechanical patterns between weather conditions may contribute to overuse running-related injuries [5]. For example, when pelvic drop was important for classification (e.g. Runner 1), there was greater pelvic drop in spring than winter, but when it was not important (e.g. Runners 2 and 3), it was lower in spring than winter. A similar pattern was observed in vertical oscillation of the pelvis: when it was important (e.g. Runners 2 and 5), there was greater amounts of oscillation in spring than winter, but when it was less important (e.g. Runner 4), there was greater oscillation in winter than spring. These results suggest that the runners involved in the current study adjusted to different weather conditions by reducing vertical or frontal plane motion accompanied by slight increases in running cadence and shorter stride length. However, it is important to note that all of the participants were injury-free and these aforementioned gait changes were not necessary to mitigate symptoms of injury. On the other hand, adopting a more constrained running pattern may, over time, may contribute to an overuse running injury [40]. Future prospective research is therefore necessary to help understand the inter-relationship between environmental weather conditions, concomitant and subject-specific changes in gait patterns, and the etiology of injury.

The RF classifier has received increasing attention within the gait-related research community due to its ability to yield excellent classification results and its fast-computational processing speed [41, 42]. In addition, this classifier provides consistent classifications using predictions derived from an ensemble of decision trees as well as a ranking of the variables according to their ability to differentiate between the target classes [41, 43]. The results of the current study are largely consistent with previous RF-based gait biomechanics studies involving wearable sensors (40,41). However, while research has investigated how IMUs systems can be used for the assessment of running biomechanics in laboratory and clinical settings [44], very few studies have been conducted in real-world settings [45, 46]. Therefore, to provide insights into this knowledge gap and open new research directions, the current study developed and evaluated subject-specific methods, using an RF classifier using data from a single IMU, and achieved excellent classification accuracy results. Interestingly, the slight differences in classification accuracy obtained between the two tested RF-methods suggest that the inclusion of information from multiple runs is beneficial for building a successful model. In addition, the current study demonstrates that the RF algorithm was able to accurately classify and determine the relative importance of each input variable for an individual runner [47, 48].

While it is important to note that the combination of multiple variables was needed to achieve a high classification accuracy and fully understand the multidimensional characteristics of the subject-specific running biomechanics associated with different weather conditions, the current findings can be compared to previous studies that have either addressed the effects of temperature on running performance [9, 49, 50] or injury rates [51]. For example, our findings are consistent with previous work demonstrating the usefulness of multidimensional analyses to better understand the complex patterns and inter-relationships between multiple biomechanical variables when classifying runners based on subtle differences in gait patterns that may be indicative of performance and/or injury [52–55]. Moreover, in the current study, regardless of the classification method, all runners exhibited slightly lower values for all biomechanical gait variables, except cadence, during winter as compared to spring. These findings support previous research indicating a more economical running technique with a lower risk of overuse injury during winter (colder) weather conditions [56–58]. Reduced pelvic drop has also been considered a protective factor for patellofemoral pain [59, 60], as well as a gait retraining strategy to reduce pain associated with this common running-related injury [61]. Future research is therefore necessary using wearable sensors in real-world situations to help better elucidate these inter-relationships.

To our knowledge, this is the first study to quantify subject-specific changes in real-world running gait biomechanics as a result of changes in environmental weather conditions. Moreover, the current study also represents one of the first investigations to analyse data from a runner’s actual training run. Specifically, a recent systematic review [62] suggested that future studies should involve long-term data collections, across multiple running bouts, and in a runner’s natural environment, thus enabling prospective studies and the development of subject-specific models of gait. Considering that the etiology of overuse running injuries is multifactorial, and can result from the interaction of many extrinsic factors such as environmental conditions, the results of the current study are an important contribution to help to better understand injury etiology.

## Limitation and future directions

The stated findings should be considered with respect to limitations. First, although there was a small number of runners (n = 6), the method employed is generalizable considering that we used subject-specific models to measure changes in gait parameters across 66,370 strides. Regardless, further investigation using a larger sample size is necessary to determine if homogenous sub-groups, or clusters, will form as a result of consistent within-group biomechanical changes (18,58). Second, we did not include any non-weather-related factors such as changes in runner’s clothing, footwear, nutrition, sleep, or daily mood state profile. Future research should consider these factors in order to gain a more complete understanding of how external factors can influence running gait biomechanics. Third, although the present study examined two different weather conditions, these results of the present study may only be applicable to these weather conditions and temperatures. As well, the temperatures in the present study (*i*.*e*., -10°C to +6°C) were lower than those of Ely et al., [9] (*i*.*e*., +5°C to +25°C) and Knapik et al., [51] (*i*.*e*., +15°C to +35°C). Lastly, a limited number of spatiotemporal and biomechanical variables obtained from a commercially available wearable sensor device were used for the current study. While it is likely that additional or more complex variables from one or more wearable sensors could improve the classification accuracy of the current study, we posit that the simplicity and translatability to the current market of wearable sensors is a significant advantage that should not be overlooked. Regardless, future research should include a broader range of variables, and possibly more wearable sensor devices, in order to gain a deeper understanding for subject-specific changes in gait patterns during out-of-laboratory data collections.

## Conclusion

In summary, our developed RF-based subject-specific classification model demonstrated excellent mean classification accuracies (87.18% and 95.42%) based on a large set of running gait data from a small group of runners. These novel results support the use of a robust machine learning approach for determining subject-specific changes in running gait patterns based on differences in external weather conditions using a single IMU device. We believe that our RF-based method may provide a more in-depth understanding of changes in gait biomechanics in response to extrinsic injury-risk factors and therefore conclude that the relationship between environmental weather conditions and gait biomechanics is subject-specific and multifactorial and involves unique interactions between intrinsic and extrinsic factors.

## Supporting information

S1 File Validation for Runner 4 (R-4) using Method 1 (one-against-another); classification accuracy: 91.42%. (XLSX)

S2 File Validation for Runner 4 (R-4) using Method 2 (partitioning datasets in two sets at a ratio of 7:3); classification accuracy: 97.56%. (XLSX)
